# Fall Detection with the Support Vector Machine during Scripted and Continuous Unscripted Activities

**DOI:** 10.3390/s120912301

**Published:** 2012-09-07

**Authors:** Shing-Hong Liu, Wen-Chang Cheng

**Affiliations:** Department of Computer Science and Information Engineering, Chaoyang University of Technology, 168, Jifong E. Rd., Wufong District, Taichung, 41349, Taiwan; E-Mail: shliu@cyut.edu.tw

**Keywords:** accelerometer, threshold-based classifier, falling detection, activities of daily life, support vector machine

## Abstract

In recent years, the number of proposed fall-detection systems that have been developed has increased dramatically. A threshold-based algorithm utilizing an accelerometer has been used to detect low-complexity falling activities. In this study, we defined activities in which the body's center of gravity quickly declines as falling activities of daily life (ADLs). In the non-falling ADLs, we also focused on the body's center of gravity. A hyperplane of the support vector machine (SVM) was used as the separating plane to replace the traditional threshold method for the detection of falling ADLs. The scripted and continuous unscripted activities were performed by two groups of young volunteers (20 subjects) and one group of elderly volunteers (five subjects). The results showed that the four parameters of the input vector had the best accuracy with 99.1% and 98.4% in the training and testing, respectively. For the continuous unscripted test of one hour, there were two and one false positive events among young volunteers and elderly volunteers, respectively.

## 1. Introduction

In 2010, 10.7% of Taiwan's population was age 65 or older. According to the government's evaluation, this proportion will become over 20% by 2025. Among the elderly, falls are not only life threatening [1] but also herald an inability to live independently [2]. Injuries from falls include broken bones, superficial cuts and abrasions to the skin as well as connective and soft tissue damage [3–5]. Detection of a fall would help to reduce the time between the fall and the arrival of medical attention [6,7], which could be realized either through an automatic fall-detection system or through a Personal Emergency Response System (PERS). However, the most common existing PERS using the push-button pendant is not always a satisfactory fall-detection method when elderly people lose consciousness or faint [8].

In recent years, the number of proposed fall-detection systems developed has increased dramatically [1,9]. The waist is a popular location for a fall-detection system because it provides reliable indications of full-body movements [10–12]. Studies have evaluated a set of fall-detection algorithms on data that recorded from 20 middle-aged volunteers (40–65 years old), performing six different falls and four scripted activities of daily living (ADL) [10,11]. The same ADLs were also recorded from 21 adults (aged 58–98 years) in a residential care unit. The results showed that a threshold on the impact and posture can achieve 97.5% sensitivity and 100% specificity. Chao *et al.* [13] recorded seven male subjects performing eight different types of falls and 17 functional ADLs. Using a combination algorithm of acceleration cross-products and post-fall postures, a sensitivity of 100% and a specificity of 49.8% were obtained. Bourke *et al.* [14] recorded 10 young healthy volunteers performing 240 falls and 120 ADLs and 10 elderly healthy volunteers performing 240 scripted ADLs; they employed thresholds on the velocity, impact and posture to achieve 100% specificity and sensitivity with a false-positive rate of 0.6 FP/day for continuous unscripted activities.

In previous studies, the signals of a tri-axial accelerometer were combined to produce many action parameters, such as the total sum vector, the dynamic sum vector, the sliding sum vector, the velocity and the tilting angle. Prior studies [14,15] have defined thresholds as criteria for fall-detection systems. A falling ADL is considered as falling down, such as forward fall, backward fall and lateral fall with both legs straight or with knee flexion [9–16]. The ADLs consist of sitting on a bed or chair, lying on a bed, walking up and down the stairs and walking [9–16]. However, the acts of standing and falling down are not classified as falling ADLs. Falling ADLs also include sitting at the bedside and slipping on the ground, sitting on a wheelchair and slipping on the ground, rolling off a bed or falling out of bed [17].

Support vector machine (SVM)-based classification methods represent a major development in pattern recognition research, for which innovation is the ability to find a hyperplane dividing samples into two classes and having the widest margin between them. Moreover, the hyperplane concept can extend a higher dimensional set with a kernel function to make a nonlinear separating hyperplane. This hyperplane, with maximum margins, can be formulated as a quadratic optimization problem in feature space. The subsets of patterns that are closest to the decision boundary are called support vectors. Therefore, SVMs have been applied to many pattern recognition and classification problems in bioinformatics [18–22]. Brown *et al.* [23] described a successful use of SVMs applied to gene expression data for the task of classifying unseen genes. Dehmeshki *et al.* [24] used SVMs for the classification of lung data. Chu *et al.* [25] applied SVMs for cancer diagnosis based on micro-array gene expression data and protein secondary structure prediction. Guler and Ubeyli [26] used SVMs on the classification of EEG signals. SVMs are also applied to ECG signal analysis and arrhythmia classification [27–29]. SVMs can also be used as criteria to detect the QRS complexes in 12-lead electrocardiograms [30].

In this study, we will first define falling ADLs as actions in which the center of gravity of the body quickly descends. These activities include, but are not limited to, slipping while ascending stairs, slipping while descending stairs, stumbling and falling down forwards, backwards falling down, lateral falling down, and falling down with a weak leg but also sitting on a bedside and slipping onto the ground, sitting in a wheelchair and slipping onto the ground, rolling down from a bed, and falling down on a bed. Moreover, the accelerometer measures the moving acceleration. If a non-falling ADL does not include the body's center of gravity falling down, then it is certain to be distinguished from the falling ADLs. Thus, our studies focus on non-falling ADLs in which the action involves the center of gravity falling. The non-falling ADLs include walking up stairs, walking down stairs, sitting down on a bed, standing up from a bed, sitting down in a wheelchair, standing up from a wheelchair, walking, lying down, sitting up from lying, squatting down, and standing up. Second, we used the hyperplane of the SVM as the separating plane to replace the traditional threshold method for the detection of falling ADLs on a comprehensive dataset containing simulated falling ADLs, non-falling ADLs, and continuously scripted ADLs, including falling ADLs and continuous unscripted ADLs performed by young and elderly volunteers with our designed device.

## 2. Overview of System

**Figure 1.**
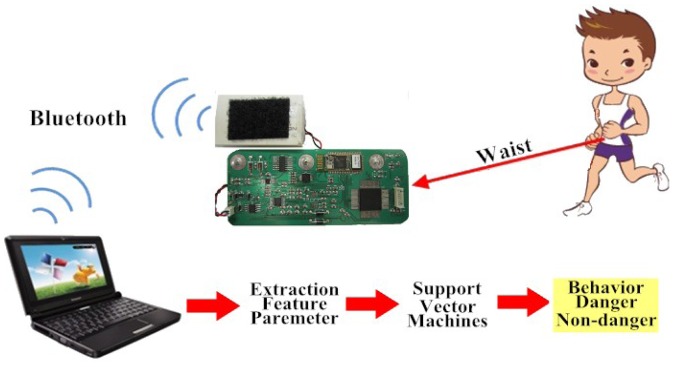
Overview of system.

Figure 1 shows the flow diagram of our method. Subjects wore our designed device at the waist, which had a tri-axial accelerometer (Kionix Inc., KXPA4-2050, Range: ±2 g), a Bluetooth module (Atrie Inc., BTM-204B), and a microprocessor (Texas Instruments Inc., MSP430 F5438). The resolution was 12 bits and the sampling rate was 200 Hz for the signals of the tri-axial accelerometer. Digital accelerometer signals were then transferred to a Bluetooth chip and transmitted wirelessly to a remote server. A Visual Basic-based interface system was used to receive a Bluetooth transmitted signal and also to display and store information. Furthermore, feature extraction and falling ADL detection were performed by a program coded in Matlab.

## 3. Feature Extraction

Four different parameters that are associated with falling ADLs were examined: total sum vector, fast changed vector, vertical acceleration, and posture angle [14–16]. First, the total sum vector, *SV_Total_*(*t*), containing both the dynamic and static acceleration components is calculated from the sampled data, as indicated below:

(1) $$SV_{\text{Total}}(t)=\sqrt{A_{x}^{2}(t)+A_{y}^{2}(t)+A_{z}^{2}(t)}$$

where *A_x_*(*t*), *A_y_*(*t*), and *A_z_*(*t*) is the acceleration in the *x*-, *y*-, and *z*-axes at time *t*, respectively.

Second, the fast changed vector, *CV_Fast_*(*t*), is calculated using the differences between the maximum and minimum acceleration in a 0.1 s sliding window for each axis, as follows:

(2) $$CV_{\text{Fast}}(t)=\sqrt{{\left(A_{x\_\max}-A_{x\_\min}\right)}^{2}+{\left(A_{y\_\max}-A_{y\_\min}\right)}^{2}+{\left(A_{z\_\max}-A_{z\_\min}\right)}^{2}}$$

where *A_max_* and *A_min_* are the maximum and minimum acceleration values in a 0.1 s sliding window. Next, the vertical acceleration, *VA*(*t*), is calculated as follows:

(3) $$VA(t)=\frac{SV_{\text{Total}}^{2}(t)-SV_{D}^{2}(t)-{\left|\vec{G}\right|}^{2}}{2\left|\vec{G}\right|}$$

where *SV_D_*(*t*) is calculated similar to the high pass filtered data, by using Equation (1), and $\vec{G}$ is the gravitational component. Finally, the Posture angle, *Φ_z_*(*t*), is defined as the angle between the *VA*(*t*) and gravity, as follows:

(4) $$\Phi_{z}(t)=\cos^{-1}(\frac{VA(t)\cdot \vec{G}}{\left|VA(t)\right|\;\left|\vec{G}\right|})\frac{180}{\pi }$$

## 4. Support Vector Machines

Considering a linearly separable dataset {**X***_i_*, *D_i_*}, *i* = 1, …, *m*, where **X***_i_* is the input pattern for the *i*th example and *D_i_* is the corresponding desired output (1, or −1), a hyperplane could be identified as the decision surface. This hyperplane can be written as follows:

(5) $$\begin{array}{l} W^{T}X_{i}+b\geq 1,\text{then}\;D_{i}=1\\ W^{T}X_{i}+b<-1,\text{then}\;D_{i}=-1 \end{array}$$

where **W** is the coefficients' vector of the hyperplane function and *b* is the distance from the origin perpendicular to the hyperplane. The margin between the hyperplane and the nearest point is maximized and can be considered as a quadratic optimization problem:

(6) $$\min\frac{1}{2}(W^{T}W)$$

(7) $$\text{subject to}\;D_{i}(W^{T}X_{i}+b)\geq 1$$

When **W** and *b* are rescaled, the point nearest to the hyperplane has a distance of 1/‖**w**‖. By the use of *Lagrange multipliers α_i_* ≥ 0, and the Kuhan–Tuker theorem, the solution is given by the following:

(8) $$W=\sum_{j\in \text{SV}}D_{j}\alpha_{j}X_{j}$$

Only a small fraction of the *α_j_* coefficient is nonzero. The corresponding pairs of **X***_j_* are known as support vectors (SV) and they define the decision boundary. All of the other input patterns with corresponding zero *α_j_* values are rendered irrelevant.

If the data are noisy, there will, in general, be no separation in the feature space. To optimize the margin slack vector, we need to introduce slack variables to allow the margin constraints to be violated. We can rewrite the objective function of the quadratic optimization problem as the following:

(9) $$\min\frac{1}{2}(W^{T}W)+C\sum_{i=1}^{m}\xi_{i}^{2}$$

(10) $$\text{subject to}\;D_{i}(W^{T}X_{i}+b)\geq 1-\xi_{i},\xi_{i}\geq 0$$

The solution given is similar to Equation (8) and the *Lagrange multipliers* are 0 ≤ *α_i_* ≤ *C*, *i* = 1, …, *m*. Finally, the hyperplane decision function for the input pattern vector **X** can be written as follows:

(11) $$f(X)=\text{sgn}(\sum_{j\in \text{SV}}D_{j}\alpha_{j}(X_{j}^{T}X)+b)$$

By replacing the inner product **X**T j**X** with the kernel function *K*(**X, X***_j_*), the input patterns are mapped to a higher dimensional space [31]. In this higher dimension, a separating hyperplane is constructed to maximize the margin.

We used LIBSVM software to detect the falling ADLs. LIBSVM is an integrated software package for support vector classification, regression and distribution estimation [32]. In the present problem, the Gaussian radial basis function was used to construct the kernel function, which is given below:

(12) $$K(X,X_{j})=\exp(-\gamma {\left\|X-X_{j}\right\|}^{2})$$

where parameter *γ* can be viewed as the radial's size, which is set at 5.3. The cost parameter *C* is 4.7.

## 5. Experimental Results

There are three experimental procedures, including simulated ADL-based young volunteers, continuous unscripted ADL without falling ADL-based young and elderly volunteers, and continuous unscripted ADL with three falling ADL-based young volunteers. The classification performances were examined based on the sensitivity, specificity, and total classification accuracy. The sensitivity is the number of true positive (TP) decisions divided by the number of actual positive cases; the specificity is the number of true negative (TN) decisions divided by the number of actual negative cases. The total classification accuracy is the number of correct decisions divided by the total number of cases.

### 5.1. Simulated ADL

In this experiment, the two-fold cross-validation was used on half of the recorded data for training and the second half of the data from the same individuals for testing. Another testing was used on half of the subjects for training and half of the subjects for testing. However, much personal equipment has to be calibrated before use for the user; therefore using the user's behavior to train personal equipment is a more feasible method.

Ten healthy young volunteers (Set 1 in Table 1), five male and five female, performed 10 simulated falling ADLs and 11 simulated non-falling ADLs. Each ADL was repeated 10 times for the first experiment. The volunteers ranged from 24 to 35 years (27.2 ± 3.6 years), with a body mass of 68–111 kg (84.27 ± 14.4 kg) and a height of 1.65 to 1.96 m (1.81 ± 0.1 m). Simulated falling ADLs and non-falling ADLs were organized and are numbered in Table 2. The height of the bed was 62 cm, and the height of the wheelchair was 50 cm.

In simulated ADLs, we randomly chose five recorded signals of every ADL for each subject as the training input. Thus, the falling ADLs have 500 datasets, and the non-falling ADLs have 550 dataset. We extracted the ADL parameters described in Section 3 from the recorded signals. The training dataset were transformed into the format of LIBSVM. A training instance matrix is an *m* by *n* matrix. In this study, *n* is the ADL parameter's number. The number of training instances was equal to the sampled number of 1,050 selected datasets. The profile of the *CV_Fast_* parameter was used to mark the falling range. We found the maximum value of the *CV_Fast_* of each falling ADL. This value multiplied by 0.87 was set as the threshold. The elements of the training label vector were set to 1 when the training instance was over this threshold and were set to −1 when it was not over the threshold or belonged to a non-falling ADL. Table 3 shows the optimal training results with different parameters' combinations. We found that the combination of four parameters has the best results, with an accuracy of 99.14%, a sensitivity of 99.60%, and a specificity of 98.73%. Therefore, we used this SVM model to test the three experimental procedures. The first phase is the test of the simulated ADL.

Another 1,050 datasets were used to test the performance of the SVM. Table 4 shows the testing of the optimal results with different parameter combinations with the same criteria in training the SVM models. We also find that the results of four parameters are best, with an accuracy of 98.38%, a sensitivity of 97.40%, and a specificity of 99.27%. In addition, the pieces of equipment used in the test include one Intel i5 M480 2.67 GHz processor, 4 GB memory and Windows 7 operating system while the Matlab software 2009 version was used for development. The computation time was mainly spent on calculating the feature vectors as well as the kernel function and weight sum of the support vector. The experimental results showed that it takes about 1ms to calculate the feature vector, while the number of support vectors is about 10% of that of the training samples. Moreover, it takes about 3 ms to calculate the kernel function, so the computation time is about 4 ms which is less than the sampling time 5 ms. Therefore, this proves that the proposed method could be effective in real applications.

To analyze what actions are easily misclassified, Table 5 shows the frequency of the false classification of each ADL and the accuracy. The action of slipping when ascending stairs has the worst classification. The reason likely has to do with the procedure of the simulated action.

**Figure 2.**
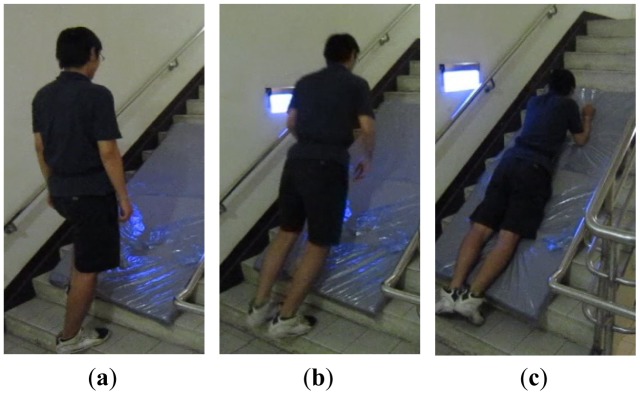
The sequence of images for the activity of slip when ascending stairs.

Figure 2 shows the decomposition of the motion, with the subject walking up the stairs, slipping and forward and falling down with a knee flexion, and the body lying on the stairs. We could find that the falling acceleration of the body's center of gravity and the falling distances are all lower than the other falling ADLs. Moreover, in continuous action including three falling ADLs, the types of missing detection are very similar to the types of worse classification in the simulated actions when the action of slipping and ascending stairs was excluded. These results show that the SVM method has a unified approach for the falling ADLs.

### 5.2. Continuous Unscripted ADL without Falling Activities

In continuous unscripted studies, five elderly volunteers, one male and four female (Set 2 in Table 1), who ranged from 70 to 83 years (74.3 ± 2.9 years), with a body mass of 46 to 65 kg (54.5 ± 4.7 kg) and a height from 148 to 158 cm (152.7 ± 2.3 cm), wore the device continuously for up to one hour. In this group, one subject had apoplexy and one subject had fallen down twice and broken his right leg bone and left humerus. During this time, volunteers conducted their normal activities, including sitting, lying, walking, walking up and down stairs, and doing house work. These studies took place at the volunteers' homes. Second, the same young volunteers (Set 1 in Table 1), including three males and two females, who executed the simulated experiment also performed continuous unscripted ADL, the same as the elderly volunteers, for up to one hour. These studies took place at the volunteers' school.

**Figure 3.**
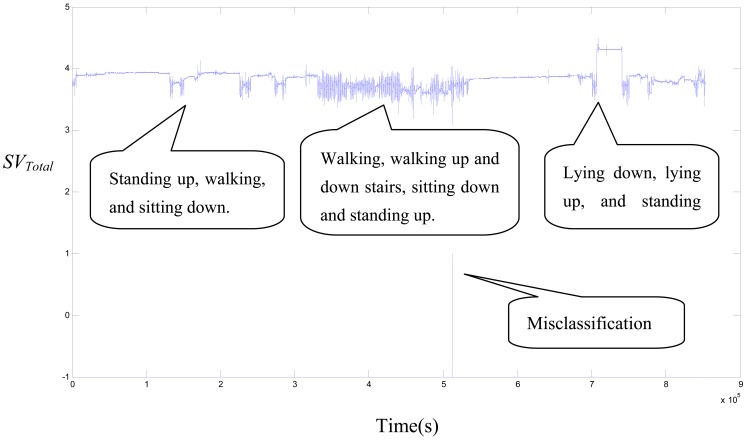
The *SV_Total_* signal of continuous actions of the EF_3 subject.

There are two conditions under which continuous action contains only non-falling ADLs for one hour. The four parameter combination in the input vector was used to test the SVM model. The false positive (FP) quantity and false positive rate (FP/h) were utilized to show the performance. The results from the same five young volunteers are shown in Table 6. The total time is 385.1 minutes and the FPs occurred three times. The false positive rate is 0.48 FP/h. The result from the five elderly volunteers is shown in Table 7. The total time is 363.8 minutes and there is one FP. The false positive rate is 0.18 FP/h. Figure 3 shows the *SV_Total_* signal for the continuous action of the EF_3 subject. There is one FP during the act of walking down the stairs. The above results show that elderly people with slow and moderate activities have a lower FP rate than young people with intense and fast activities. On the other hand, the falling definition that we detected in this paper is different from that of previous studies. Therefore, the FP rate we obtained is relatively high than the threshold-based algorithm in unscripted continuous experiments.

### 5.3. Continuous Unscripted ADL

In continuous action, including three times falling ADLs, another ten young volunteers conducted this experiment (Set 3 in Table 2). Five males and five females, who ranged from 24 to 28 years (26.4 ± 2.2 years), with a body mass of 68–111 kg (84.27 ± 14.4 kg) and a height from 170 to 176 cm (173.8 ± 1.1 cm), continued to wear the device for 5 minutes. They not only conducted the non-falling ADLs, like the elderly volunteers, but also conducted falling ADLs three times during this period.

**Figure 4.**
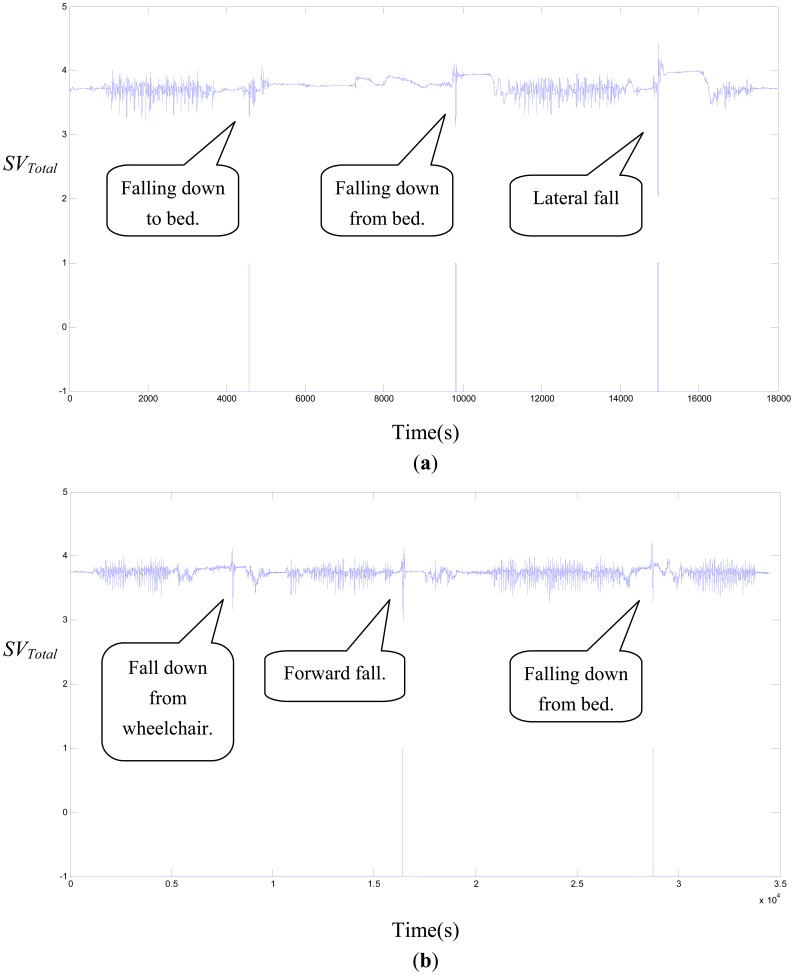
The *SV_Total_* signal for the continuous action of the (**a**) CM_3 and (**b**) CM_1 subjects.

Because the action of slipping and ascending stairs is a more difficult action, we only conducted nine falling ADLs. The results show, in Table 8, that FP is zero, but there are three false negatives (FN). We analyzed the TP and TN of each falling ADL in Table 9. Falling down from a wheelchair, rolling down from a bed, and falling down to a bed all have one missing detection. Figure 4(a) shows the *SV_Total_* signal for the continuous action of the CM_3 subject whose falling ADLs were falling down to a bed, falling down from a bed, and lateral falling down; these falling ADLs were all correctly detected. Figure 4(b) shows the *SV_Total_* signal for the continuous action of the CM_1 subject whose falling ADLs were falling down from a wheelchair, forward falling down, and falling down from a bed; these falling ADLs had one missing detection.

### 5.4. The Comparison with the Threshold-Based Algorithm

**Figure 5.**
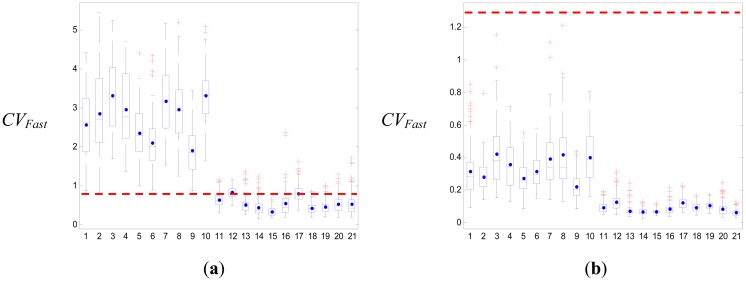
The boxplots of the *CV_Fast_* parameter for all of the ADLs. (**a**) The upper peak values of the *CV_Fast_* signal, for which the upper falling threshold is 0.8. (**b**) The lower peak values of the *CV_Fast_* signal, for which the upper falling threshold is 1.26.

**Figure 6.**
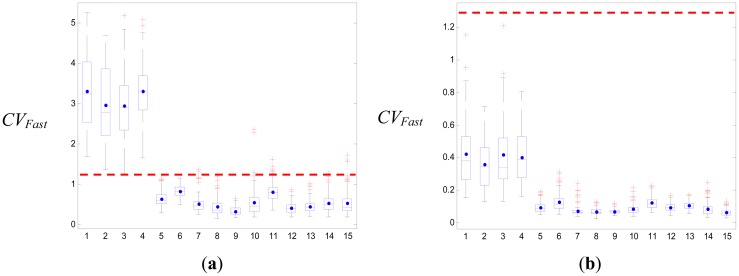
The boxplots of the *CV_Fast_* parameter for only four falling ADLs. (**a**) The upper peak values of the *CV_Fast_* signal, for which the upper falling threshold is 1.25. (**b**) The lower peak values of the *CV_Fast_* signal, for which the upper falling threshold is 1.29.

Previous studies have frequently used a threshold-based algorithm with the impact and posture after the fall as the most popular method for fall detection [10,11,13–16]. The falling ADLs are described by calculating an action's parameters, such as the SV_Total_, CV_Fast_, VA, *Φ*_z_, or velocity, and defining their upper and lower falling thresholds with respect to the impact and the posture. A boxplot (see Figure 5 or Figure 6) was used to set the thresholds of these parameters. Moreover, previous studies have focused on the actions of standing and falling down as the falling ADLs. We know that these falling actions are all encompassed in the body's center of gravity as it quickly falls down, and the falling distances are the highest compared to the non-falling ADLs. Thus, the upper or lower fall threshold could be used to separate the falling ADL and the non-falling ADL, for which the sensitivity and specificity all are 100%. However, the falling ADLs must also include sitting on a bedside and slipping onto the ground, sitting in a wheelchair and slipping onto the ground, rolling down from a bed, or falling down to a bed. The falling acceleration of the body's center of gravity in these falling ADLs is not larger than the actions of standing and falling down, and the falling distances are close to the moving distances of the non-falling ADLs. Under these conditions, the threshold-based method is not expected to have very good results.

We used boxplots to define the upper and lower falling thresholds of four parameters that were used to separate the falling ADLs and the non-falling ADLs. The results of the simple threshold-based method in our study are shown in Table 10. The best threshold was the upper falling threshold of *CV_Fast_*, of which the specificity was 81.9%. Figure 5 shows the boxplot of *CV_Fast_*; the upper and lower falling thresholds were 0.8 and 1.26, respectively. If we considered the upper and lower falling thresholds of all the parameters, the specificity only reached 82.5%. They were much lower than our proposed SVM method. If we only considered forward falling down, backward falling down, literal falling down and falling down with leg weakness as falling ADLs, then the results of the SVM could attain an accuracy of 100% in the training and testing. The simple threshold-based method was used to detect these falling actions, with the results shown in Table 11. The best threshold was the upper falling threshold of *CV_Fast_*, of which the specificity was 98.5% and the whole specificity was 98.6%. This result was very close to the results of previous studies. However, the result was also lower than the SVM method. Figure 6 shows the boxplot of *CV_Fast_*; the upper and lower falling thresholds were 1.25 and 1.29, respectively.

## 6. Conclusions

We used the hyperplane of the SVM as the threshold for detecting the 21 ADLs, including 10 falling ADLs and 11 non-falling ADLs. The most successful parameter combination, *SV_Total_* + *CV_Fast_* + *VA* + *Φ_z_*, achieved a training sensitivity and specificity of 99.60% and 98.73%, and a testing sensitivity and specificity of 97.40% and 99.27%, respectively If the threshold-based method was used to detect the falling ADLs, then the specificity was only 82.5%. Thus, the conclusion is that when the parameters of falling and non-falling ADLs are very close, the results of the SVM method are better than the threshold-based algorithm. In unscripted continuous experiments, the FP rates were 0.48 FP/h, 0.18 FP/h and 0 FP/h for the same young volunteers, for elderly volunteers and for different young volunteers, respectively. In continuous action, including three times falling ADLs, the sensitivity also became 90%. On the other hand, the falling definition that we detected in this paper is different from that of previous studies. Therefore, the FP rate we obtained is relatively high than the threshold-based algorithm in unscripted continuous experiments.

## Figures and Tables

**Table 1.** Information on the volunteers for all of the experiments.

**Set**	**No.**	**Gender**	**Age (year)**	**High (cm)**	**Weight (kg)**	**BMI**

Set 1	M_1	Male	22	182	73	22.5
	M_2	Male	23	178	73	21.7
	M_3	Male	24	187	80	22.8
	M_4	Male	24	178	73	23.0
	M_5	Male	24	173	98	32.7
	F_6	Female	25	160	50	19.5
	F_7	Female	24	168	53	18.7
	F_8	Female	25	167	55	19.7
	F_9	Female	32	163	50	18.8
	F_10	Female	17	155	47	19.5

Set 2	EF_1	Female	70	155	46	19.1
	EF_2	Female	71	150	65	28.9
	EF_3	Female	83	148	47	21.5
	EF_4	Female	73	158	60	24.0
	EM_5	Male	71	178	80	25.2

Set 3	CM_1	Male	28	175	79	25.8
	CM_2	Male	28	173	60	20.0
	CM_3	Male	28	175	68	22.2
	CM_4	Male	24	170	95	32.9
	CM_5	Male	24	176	88	28.4
	CF_6	Female	19	158	50	20.0
	CF_7	Female	18	160	48	18.8
	CF_8	Female	19	162	52	19.8
	CF_9	Female	19	165	55	20.2
	CF_10	Female	22	155	71	29.6

**Table 2.** A series of falling and non-falling ADLs.

**Falling ADLs**		**Non-Falling ADLs**	

No.	Action	No.	Action
1	Slip and ascending stairs	11	Ascending stairs
2	Slip and descending stairs	12	Descending stairs
3	Forward fall	13	Sitting down on bed
4	Backward fall	14	Standing up from bed
5	Falling down from bed	15	Sitting down in wheelchair
6	Fall down from wheelchair	16	Standing up from wheelchair
7	Rolling down from bed	17	Walking
8	Lateral fall	18	Lying down
9	Falling down to bed	19	Lying up
10	Fall for the weak leg	20	Squatting down
		21	Standing up

**Table 3.** The training results with different parameter combinations.

**Parameters**	**Accuracy (%)**	**Sensitivity (%)**	**Specificity (%)**

*SV_Total_, CV_Fast_*, *VA*, *Φ_z_*	99.14	99.60	98.73
*SV_Total_, CV_Fast_*, *VA*,	93.43	100.00	87.45
*SV_Total_, CV_Fast_*, *Φ_z_*	88.76	100.00	78.55
*SV_Total_, VA*, *Φ_z_*	94.10	91.60	96.36
*CV_Fast_*, *VA*, *Φ_z_*	98.95	99.40	98.55
*SV_Total_, CV_Fast_*	96.38	98.80	94.18
*SV_Total_, VA*	61.81	19.80	100.00
*SV_Total_, Φ_z_*	68.10	33.00	100.00
*CV_Fast_*, *VA*	79.71	57.40	100.00
*CV_Fast_*, *Φ_z_*	83.81	100.00	69.09
*VA*, *Φ_z_*	88.00	75.60	99.27

**Table 4.** The testing results with different parameter combinations.

**Parameters**	**Accuracy (%)**	**Sensitivity (%)**	**Specificity (%)**

*SV_Total_, CV_Fast_*, *VA*, *Φ_z_*	**98.38**	**97.40**	**99.27**
*SV_Total_, CV_Fast_*, *VA*,	90.19	99.80	81.45
*SV_Total_, CV_Fast_*, *Φ_z_*	83.52	99.80	68.73
*SV_Total_, VA*, *Φ_z_*	90.19	93.20	87.45
*CV_Fast_*, *VA*, *Φ_z_*	98.10	98.00	98.18
*SV_Total_, CV_Fast_*	97.33	99.00	95.82
*SV_Total_, VA*	60.67	17.60	99.82
*SV_Total_, Φ_z_*	69.81	36.60	100.00
*CV_Fast_*, *VA*	83.24	65.00	99.82
*CV_Fast_*, *Φ_z_*	82.95	64.80	99.45
*VA*, *Φ_z_*	93.24	89.20	96.91

**Table 5.** The frequency of the false classification of each ADL.

**Falling ADL**			**Non-Falling ADL**		

**Action**	**# of False**	**False Rate**	**Action**	**# of False**	**False Rate**

Slip and ascending stairs	4	0.8%	Ascending stairs	0	0.0%
Slip and descending stairs	2	0.4%	Descending stairs	2	0.4%
Forward fall	0	0.0%	Sitting down on bed	0	0.0%
Backward fall	1	0.2%	Standing up from bed	1	0.2%
Falling down from bed	0	0.0%	Sitting down in wheelchair	0	0.0%
Fall down from wheelchair	2	0.4%	Standing up from wheelchair	0	0.0%
Rolling down from bed	2	0.4%	Walking	0	0.0%
Lateral fall	0	0.0%	Lying down	0	0.0%
Falling down to bed	2	0.4%	Lying up	0	0.0%
Fall for the weak leg	0	0.0%	Squatting down	1	0.2%
			Standing up	0	0.0%

**Table 6.** The FP number for the subjects of Table 1 Set 1 in continuous unscripted ADL without falling activities in the experiment.

**Volunteers**	**Time (min)**	**FP**

F_6	79.1	0
M_3	74.8	1
M_5	84.2	0
F_7	67.6	2
M_2	79.4	0
Total	385.1	3

**FP Rate (FP/h)**		0.48

**Table 7.** The FP number for the subjects of Table 1 Set 2 in the continuous unscripted ADL without falling activities in the experiment.

**Volunteers**	**Time (min)**	**FP**

EF_1	72.1	0
EF_2	71.1	0
EF_3	76.2	1
EF_4	76.3	0
EM_5	68.1	0
Total	363.8	1

**FP Rate (FP/h)**		0.18

**Table 8.** The statistics of falling ADL detection for subjects of Table 1 Set 3 in the continuous unscripted ADL experiment.

**Volunteers**	**TP**	**FN**	**FP**

CM_1	2	1	0
CM_2	3	0	0
CM_3	3	0	0
CM_4	2	1	0
CM_5	3	0	0
CF_6	3	0	0
CF_7	2	1	0
CF_8	3	0	0
CF_9	3	0	0
CF_10	3	0	0

**Total**	**27**	**3**	**0**

Sensitivity		90%	

**Table 9.** The analysis of different falling ADL detections in a continuous unscripted ADL experiment.

**Falling ADL**	**Numbers**	**TP**	**FN**

Slip and descending stairs	2	2	0
Forward fall	4	4	0
Backward fall	3	3	0
Lateral fall	3	3	0
Falling down from bed	3	3	0
Fall down from wheelchair	3	2	1
Rolling down from bed	2	1	1
Fall for the weak leg	6	6	0
Falling down onto bed	4	3	1

**Total**	**30**	**27**	**3**

**Table 10.** The testing results of threshold-based methods for all of the ADLs.

**Parameters**	**Specificity of Upper Threshold (TN)**	**Specificity of Lower Threshold (TN)**

*SV_Total_*	0%	5.2%
*CV_Fast_*	81.9%	0%
*Φ_z_*	4.6%	6.7%
*VA*	0%	0%

**Total specificity**	**82.5%**	

**Table 11.** The testing results of the threshold-based method for only four falling ADLs.

**Parameters**	**Specificity of Upper Threshold (TN)**	**Specificity of Lower Threshold (TN)**

*SV_Total_*	0.00%	5.2%
*CV_Fast_*	98.5%	0.00%
*Φ_z_*	4.6%	29.9%
*VA*	0.00%	0.00%

**Total specificity**	**98.6%**	

## References

[1] Ozcan A., Donat H., Gelecek N., Ozdirenc M., Karadibak D. (2005). The relationship between risk factors for falling and the quality of life in older adults. BMC Public. Health.

[2] Yardley L., Smith H. (2002). A prospective study of the relationship between feared consequences of falling and avoidance of activity in community-living older people. Gerontologist.

[3] Salva A., Bolibar I., Pera G., Arias C. (2004). Incidence and consequences of falls among elderly people living in the community. Med. Clin..

[4] Baker S.P., Harvey A.H. (1985). Fall injuries in the elderly. Med. Clin. Geriatr..

[5] Tinetti M.E., Williams C.S. (1997). Falls injuries due to falls, and the risk of admission to a nursing home. N. Engl. J. Med..

[6] Doughty K., Lewis R., McIntosh A. (2000). The design of a practical and reliable fall detector for community and institutional telecare. J. Telemed. Telecare.

[7] Huang C.-N., Chiang C.-Y., Chen G.-C., Hsu S., Chu W.-C., Chan C.-T. (2010). Fall detection system for healthcare quality improvement in residential care facilities. J. Med. Biol. Eng..

[8] Gurley R.J., Lum N., Sande M., Lo B., Katz M.H. (1996). Persons found in their homes helpless or dead. N. Engl. J. Med..

[9] Yang C.C., Hsu Y.L. (2010). A review of accelerometry-based wearable motion detectors for physical activity monitoring. Sensors.

[10] Kangas M., Konttila A., Lindgren P., Winblad I., Jamsa T. (2008). Comparison of low-complexity fall detection algorithms for body attached accelerometers. Gait Posture.

[11] Bourke A.K., O'Brien J.V., Lyons G.M. (2007). Evaluation of a threshold-based tri-axial accelerometer fall detection algorithm. Gait Posture.

[12] Liu S.-H., Chang Y.-J. (2009). Using accelerometers for physical actions recognition by a neural fuzzy network. Telemed. J. e-Health.

[13] Chao P.-K., Chan H.-L., Tang F.-T., Chen Y.-C., Wong M.-K. (2009). A comparison of automatic fall detection by the cross-product and magnitude of tri-axial acceleration. Physiol. Meas..

[14] Bourke A.K., Van de Ven P., Gamble M., O'Connor R., Murphy K., Bogan E., McQuade E., Finucane P., O′Laighin G., Nelson J. (2010). Evaluation of waist-mounted tri-axial accelerometer based fall-detection algorithms during scripted and continuous unscripted activities. J. Biomech..

[15] Kangas M., Vikman I., Wiklander J., Lindgren P., Nyberg L., Jamsa T. (2009). Sensitivity and specificity of fall detection in people aged 40 years and over. Gait Posture.

[16] Bourke A.K., Lyons G.M. (2008). A threshold-based fall-detection algorithm using a bi-axial gyroscope sensor. Med. Eng. Phys..

[17] Lamb S., Jørstad-Stein E.C., Hauer K., Becker C. (2005). Prevention of falls network Europe and outcomes consensus group. Development of a common outcome data set for fall injury prevention trials: The prevention of falls network Europe consensus. J. Am. Geriatr. Soc..

[18] Kecman V. (2001). Learning and Soft Computing, Support Vector Machines, Neural Networks and Fuzzy Logic Models.

[19] Wang L.P. (2005). Support Vector Machines: Theory and Application.

[20] Sloin A., Burshtein D. (2008). Support vector machine training for improved hidden markov modeling. IEEE Trans. Signal Process.

[21] Wang L.P., Fu X.J. (2005). Data Mining with Computational Intelligence.

[22] Khandoker A.H., Palaniswami M., Karmakar C.K. (2009). Support vector machines for automated recognition of obstructive sleep apnea syndrome from ECG recordings. IEEE Trans. Inf. Technol. Biomed..

[23] Brown M., Grundy W., Lin D., Cristianini N., Sugnet C., Furey T. (2000). Knowledge based analysis of micro-array gene expression data using support vector machine. Proc. Natl. Acad. Sci. USA.

[24] Dehmeshki J., Chen J., Casique M.V., Karakoy M. Classification of Lung Data by Sampling and Support Vector Machine.

[25] Chu F., Jin G., Wang L. (2005). Cancer diagnosis and protein secondary structure prediction using support vector machine. Stud. Fuzz. Soft Comput..

[26] Guler I., Ubeyli E.D. (2007). Multiclass support vector machines for EEG-signals classification. IEEE Trans. Inf. Technol. Biomed..

[27] Osowski S., Hoai L.T., Markiewicz T. (2004). Support vector machines based expert system for reliable heartbeat recognition. IEEE Trans. Biomed. Eng..

[28] Acir N. (2005). Classification of ECG beats by using a fast least square support vector machines with a dynamic programming feature selection algorithm. Neural Comput. Appl..

[29] Acir N. (2006). A support vector machine classifier algorithm based on a perturbation method and its application to ECG beat recognition systems. Expert Syst. Appl..

[30] Mehta S.S., Lingayat N.S. (2009). Identification of QRS complexes in 12-lead electrocardiogram. Expert Syst. Appl..

[31] Burges C.J.C. (1998). A tutorial on support vector machines for pattern recognition. Data Min. Knowl. Discov..

[32] Chang C.-C., Lin C.-J. (2004). LIBSVM: A Library for Support Vector Machines.

